# Protease-armed bacteria in the skin

**DOI:** 10.1007/s00441-012-1355-2

**Published:** 2012-02-23

**Authors:** Joanna Koziel, Jan Potempa

**Affiliations:** 1 Department of Microbiology, Faculty of Biochemistry, Biophysics and Biotechnology, Jagiellonian University, ul. Gronostajowa 7, 30-387 Kraków, Poland; 2 Oral Health and Systemic Diseases Research Group, University of Louisville School of Dentistry, Louisville, KY USA

**Keywords:** Protease, Skin, Host defense

## Abstract

The skin constitutes a formidable barrier against commensal and pathogenic bacteria, which permanently and transiently colonise the skin, respectively. Commensal and pathogenic species inhabiting skin both express proteases. Whereas proteases secreted by commensals contribute to homeostatic bacterial coexistence on skin, proteases from pathogenic bacteria are used as virulence factors, helping them colonise skin with breached integrity of the epithelial layer. From these initial sites of colonisation, pathogens can disseminate into deeper layers of skin, possibly leading to the spread of infection. Secreted bacterial proteases probably play an important role in this process and in the deterrence of innate defence mechanisms. For example, *Staphylococcus aureus* proteases are essential for changing the bacterial phenotype from adhesive to invasive by degrading adhesins on the bacterial cell surface. Secreted staphylococcal proteases mediate pathogen penetration by degrading collagen and elastin, essential components of connective tissue in the dermis. The activation of the contact system and kinin generation by *Streptococcus pyogenes* and *S. aureus* proteases contributes to an inflammatory reaction manifested by oedema, redness and pain. Kinin-enhanced vascular leakage might help bacteria escape into the circulation thereby causing possible systemic dissemination of the infection. The inflammatory reaction can also be fueled by the activation of protease-activated receptors on keratinocytes. Concomitantly, bacterial proteases are involved in degrading antimicrobial peptides, disarming the complement system and neutrophils and preventing the infiltration of the infected sites with immune cells by inactivation of chemoattractants. Together, this provides protection for colonising and/or invading pathogens from attack by antibacterial forces of the skin.

## Skin: structure and function

Skin is the largest organ in the body and acts as a physical barrier against the external environment. In addition to protecting the body against microorganisms, ultraviolet radiation, toxins, allergens or mechanical insults, skin also regulates the transport of water, electrolytes and some supplements, plus body temperature and metabolism. Skin has four structural layers: the epidermis, basement membrane, dermis and a fat layer, also called the subcutaneous layer (subcutis).

The epidermis, the outer layer of skin, is a dynamic structure composed of keratinocytes, melanocytes, Langerhans cells and Merkel cells. The mature human epidermis consists in four layers (from the innermost layer to the surface): the stratum basale (SB), stratum spinosum (SS), stratum granulosum (SG) and stratum corneum (SC). The SB, which lies adjacent to the dermis, comprises mainly dividing undifferentiated keratinocytes, which are attached to the basement membrane by hemidesmosomes. Scattered throughout the basal layer of the epidermis are pigment (melanin)-producing melanocytes. Merkel cells are also found in the basal layer in large numbers at touch-sensitive sites, such as the fingertips and lips. They are closely associated with cutaneous nerves and seem to be involved in light touch sensation. As basal keratinocytes move towards the outer layer of skin, they become connected by desmosomes, initially forming the SS and then they undergo terminal differentiation at the SG and SC layers (Kawakubo et al. 2011). The keratinocyte plasma membrane is replaced with an insoluble protein envelope, inducing aggregation of keratin intermediate filaments via filaggrin. The collapse of their cytoskeleton into flattened squames and the orientation of the keratin proteins add strength to the SC. The resulting non-viable cornifed cells, known as corneocytes and surrounded by lipids, provide the natural physical and water-retaining barrier of the skin (Candi et al. 2005). In most areas of the skin, there are 10-30 layers of dead cells. During terminal differentiation and cornification, granular keratinocytes secrete structural and adhesion proteins, lipids, antimicrobial peptides (AMPs), proteases and their inhibitors via lamellar granules into the extracellular space.

The dermis is the deepest layer of the skin and provides structural support for the skin. This layer is anchored to the epithelium by the basement membrane, a multilayered structure forming the dermo-epidermal junction. The dermis consists in fibroblasts, which produce collagen, elastin, fibrillin and structural proteoglycans, together with immunocompetent mast cells and macrophages. Collagen fibers constitute 70% of the dermis, providing strength and toughness. Elastin maintains normal elasticity and flexibility, whereas proteoglycans provide viscosity and hydration. The fibrous tissue of the dermis anchors blood and lymphatic vessels, nervous cells and fibers, sweat and sebaceous glands, follicles and small quantities of striated muscle. The subcutis or hypodermis, which is made up of a loose connective tissue layer and is an important depot of fat, lies below the dermis.

## Immune components of the skin

Apart from acting as a physical barrier between the host and the environment, the skin also plays a key immunological role in sensing and responding to invading pathogens. The skin immune system can provide early innate immune responses, which promote cutaneous inflammation and adaptive immune responses that lead to an immunological memory that can recognise foreign antigens (Kupper and Fuhlbrigge 2004). A network of endogenous proteolytic enzymes and their inhibitors play an important role in this process (Yoshida 2010; Blaber et al. 2010; Beaufort et al. 2010; Sotiropoulou and Pampalakis 2010; Ohler et al. 2010).

The constitutive innate immune mechanisms in the skin consist in (1) commensal microorganisms that occupy niches suitable for bacterial growth and (2) the corneal layer comprised of dead keratinocytes and providing the physical barrier of the skin and chemical defence in the form of AMPs. In humans, such peptides include β-defensin 2, β-defensin 3, cathelicidin and RNase 7, which are induced in response to infection and exert bacteriostatic or bactericidal activity against invading pathogens (Schauber and Gallo 2009). Keratinocytes that compose the deeper layers of the skin (granular, spinous and basal layers) express pattern recognition receptors. These include Toll-like receptors and nucleotide-binding oligomerisation domain proteins, which sense the pathogen-associated molecular patterns of invading microorganisms and initiate early cutaneous immune responses (Kawai and Akira 2010). In addition to keratinocytes, other cells contribute to the cutaneous immune responses, including Langerhans cells and γδ T cells (mice only) in the epidermis, plus macrophages, dendritic cells, mast cells, B and T cells, plasma cells, natural killer cells and fibroblasts in the dermis (Wehner et al. 2011; Tobin et al. 2011; Kupper and Fuhlbrigge 2004).

## Microflora of the skin

The skin is a habitat for commensal bacteria, including *Staphylococcus*, *Micrococcus* and *Corynebacterium* sp., which act as a barrier against colonisation by potentially pathogenic microbes and any overgrowth of opportunistic pathogens already present on the skin. Protection is exerted passively by depleting available nutrients for competing bacteria and by preventing their adherence and/or translocation across skin layers. Invaders are also actively deterred by bacteriocin (e.g. epidermin, Pep5 and epilancin K7, bacteriocins secreted by *S. epidermidis*; Bastos et al. 2009) and toxic metabolites produced by commensal microflora. Finally, the pathogenicity of invading species can be tempered by degradation of their virulence factors via proteases released by commensals. In addition to providing direct protection against pathogens, commensals promote endogenous antibacterial activity by stimulating the production of AMPs via keratinocytes and by enhancing the antibody production of immune cells and bacterial clearance by local phagocytes. Commensals also help maintain inflammatory homeostasis by suppressing excess cytokine release after minor epidermal injury.

Apart from benign commensals, the human skin is often colonised by opportunistic pathogens. Among them, *Staphylococcus aureus* and *Streptococcus pyogenes* are the most common species. Because of the frequency of these Gram-positive bacteria on healthy skin, *S. aureus* and *S. pyogenes* are sometimes considered to be part of the normal bacterial flora of the skin. This is not the case for Gram-negative organisms such as *Pseudomonas aeruginosa*, *Pasteurella multocida*, *Capnocytophaga canimorsus*, *Bartonella* sp., *Klebsiella rhinoscleromatis* and *Vibrio vulnificus*, which are never found in the normal microflora of healthy skin and are responsible for cutaneous infections (Chiller et al. 2001).

## Bacterial proteases in the skin

**Fig. 1:**
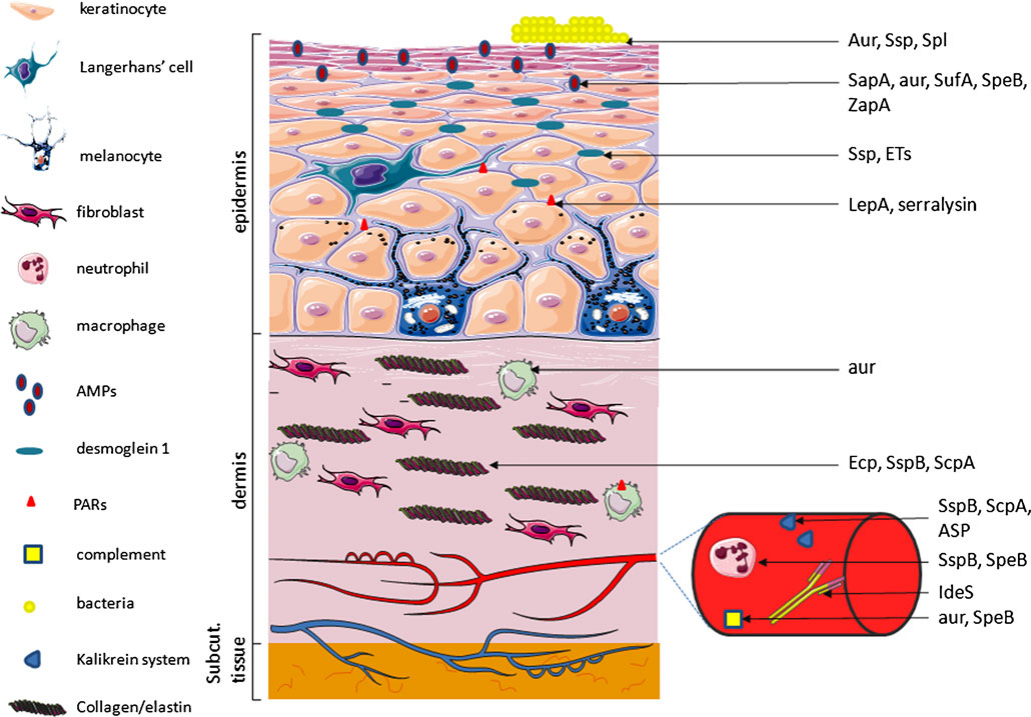
Representation of some bacterial protease targets in the skin. Bacterial proteases (*right*) contribute to skin colonisation by microorganisms by providing nutrients and by modulating bacterial adherence properties. In epidermis, bacterial proteases can neutralise antibacterial peptides (antimicrobial peptides [*AMPs*], such as LL37), creating a safe niche for AMP-susceptible pathogens. The disruption of desmosomal kadherins (desmoglein 1), which provide cell-cell adhesion, causes exfoliation in the stratum granulosum. Bacterial proteolytic activity induces protease-activated receptor (*PAR*) signalling involved in cutaneous inflammation. Pathogen penetration is facilitated by degradation of collagen and elastin, essential components of connective tissue of the dermis, by secreted bacterial proteases. Proteases produced by skin bacteria modulate the skin immune system. Professional phagocyte functions are disabled upon bacterial proteases. Proteases may also lead to the depletion of functional neutrophils at infection sites, thus facilitating pathogen colonisation and spreading in subepithelial tissues. The cleaving of antibodies by bacterial proteases leads to the avoidance of an immunological response. Bacterial proteases target and subvert the complement system by degrading or binding complement components to prevent activation of the pathway. As potent activators of the contact system, bacterial proteases may participate in the uncontrolled generation of proinflammatory mediators, inducing an excessive inflammatory reaction, which can lead to serious tissue damage (*Subcut.* subcutaneous). Figures were produced using Servier Medical Art

A variety of proteases are expressed by skin microflora, including enzymes produced by commensal species that sustain habitat homeostasis and those exploited and used as powerful virulence factors by pathogens during infection and skin injury (Fig. 1, Table 1).

**Table 1:** Bacterial proteases and their characteristics (*AMPs* antimicrobial peptides, *FNBP* fibronectin-binding protein, *HF* Hageman factor, *IL* interleukin, *PAR* protease-activated receptor, *PK* plasma prekalikrein, *PMN* polymorphonuclear leukocytes, *SSSS* staphylococcal scaled-skin syndrome, *VL* vascular leakage, *MEROPS* MEROPS peptidase database http://www.sanger.ac.uk/resources/databases/merops.html)

Bacteria	Protease	MEROPS data (MEROPS ID/protease name/protease family)	Target	Process
*Staphylococcus epidermidis*	SepA	M04.009/aureolysin/M4	AMPs	Colonisation
	Ecp	C47.003/ecp paptidase/C47	Elastin; fibrinogen and fibronectin	Connective tissue destruction
*Staphylococcus aureus*	Ssp (V8), serine protease	S01.269/glutamyl peptidase I/S1	FNBP	Bacterial adhesion
			Desmoglein 1	Cell–cell adhesion
	Aureolysin (aur), metalloprotease	M04.009/aureolysin/M4	Surface proteins Atl, Bap and SasG	Biofilm
			LL37	Colonisation
			Target unknown	Intracellular survival
			C3b; C5a	Complement inactivation
	Epidermolytic toxins (ETs)	S01.270/exfoliatin A/S1	Desmoglein 1	Cell–cell adhesion, SSSS
	Spl	S01.503;S01.282;S01.283/SplA;SplB;SplC/S1	Surface proteins Atl, Bap and SasG	Biofilm
	Staphopain B (SspB), cystein protease	C47.002/staphopain B/C47	CD11b; CD31	PMN
			Chemerin	Chemotaxis
			Cystatins: C, D, E/M	
			Elastin	Connective tissue destruction
			Kininogens	VL
	Staphopain A (ScpA), cystein protease	C47.001/staphopain A/C47	Kininogens	VL
			Cystatins: C, D, E/M	
			Elastin	Connective tissue destruction
*Finegoldia magna*	Subtilisin-like serine protease (SufA)	S08.138/SufA peptidase/S8	LL37	Colonisation
			MIG/CXCL9	Chemotaxis
*Streptococcus pyogenes*	Streptopain (SpeB), streptococcal pyrogenic exotoxin B	C10.001/streptopain/C10	LL37	Colonisation
			Mitochondria	Inactivation of PMN mitochondria
				Intracellular survival
			C3; properdin	Complement system inhibition
	Streptolysin S	-	E-cadherin	Skin penetration
	IdeS	C66.001/IdeS peptidase/C66	IgG	Opsonophagocytosis
	ScpC	S08.027/cell envelope proteinase A/S8	IL-8	
*Propionibacterium acnes*	Exogenous proteases	-	PAR-2	
*Serratia marcescens*	Serralysin	M10.051/serralysin/M10	PAR-2	
*Pseudomonas aeruginosa*	LepA	-	PAR-1, -2, -4 IL-8 secretion	PMN infiltration
	Metalloproteinase elastase	M04.005/pseudolysin/M4	Elastin; fibrinogen and fibronectin	Connective tissue destruction
			HF and PK activation	VL
			LL37	Colonisation
*Enterococcus faecalis*	Gelatinase	M04.007/coccolysin/M4	LL37	Colonisation
*Proteus mirabilis*	Metalloprotease (ZapA)	M10.057/mirabilysin/M10	LL37	Colonisation
*Aeromonas sobria*	Serine protease (ASP)	S08.125/ASP (Aeromonas sobria)-type peptidase/S8	PK	VL
*Vibrio vulnificus*	Metalloprotease	-	HF and PK activation	VL

### Role of proteases in survival and colonisation

Commensal skin microflora and pathogenic species are both well equipped with factors that promote colonisation and persistence in the harsh conditions of the skin. A crucial step that ensures successful colonisation is bacterial adherence to the horny epidermal layer. *S. aureus* express a distinct array of receptors that recognise different human extracellular matrix proteins. These receptors are termed “microbial surface components recognising adhesive matrix molecules” (MSCRAMMs; Patti et al. 1994; Foster and Hook 1998). Microbial cell surface proteins are important virulence factors and specifically bind to extracellular matrix proteins such as fibronectin (fibronectin-binding proteins, FNBPA and FNBPB), fibrinogen (clumping factors, such as ClfA and ClfB) and collagen (collagen-binding protein). They are expressed in a coordinated and sequential fashion that depends on bacterial cell density. Regulatory loci, such as accessory gene regulator (*agr*) and staphylococcal accessory regulator (*sar*), coordinate the gene expression of most *S. aureus* MSCRAMMs and secreted proteins (Novick 2003). *S. aureus* adhesins are expressed during the early exponential growth phase, when bacterial cell density is low, whereas hemolysins, toxins and enzymes facilitating tissue destruction and dissemination, including proteases, are expressed at the end of the exponential phase and during the stationary phase of growth (Pragman and Schlievert 2004). During colonisation, staphylococcal proteases play an important role by controlling the stability and/or processing of cell surface proteins. One clear example is the proteolysis of FNBP by Ssp (V8), a staphylococcal serine protease, which decreases the adhesive phenotype of *S. aureus* and allows dissemination of the pathogen (McGavin et al. 1997).

The current paradigm suggests that tissue colonisation occurs via the formation of multicellular surface-attached agglomerations of microorganisms called “biofilm”. Primary attachment of bacteria to the surface and subsequent aggregation can initiate biofilm formation, which provides resistance against many antibiotics and host defence mechanisms. In the mature biofilm, channels are formed for nutrient delivery to all biofilm cells, whereas the detachment of cell clusters can lead to the dissemination of infection. Distractive forces are crucial for both these aspects (O'Toole et al. 2000). Intracellular aggregation in biofilm is mediated by a variety of surface macromolecules, including proteins. Several lines of evidence indicate that *S. aureus* proteases are involved in the enzymatic degradation of proteinaceous biofilm formation supporting factors (Boles and Horswill 2008). The activity of *S. aureus* extracellular proteases, including the metalloprotease aureolysin (Aur) and Spl protease, has been demonstrated to be required for *agr*-mediated detachment in established biofilms. The targets of these *agr*-controlled proteases are not known but surface adhesins are likely substrates. Candidates include the surface proteins Atl, Bap and SasG, all of which play significant roles in biofilm formation (Corrigan et al. 2007; Trotonda et al. 2005; Cucarella et al. 2001; Biswas et al. 2006). Recently, the Bap protein has been identified as an Aur and SspA substrate (Martí et al. 2010). Atl is additionally known to require proteolytic processing for activation, which is inhibited by phenylmethylsulfonyl fluoride (Oshida et al. 1995). As described earlier, other possible targets of staphylococcal proteases include MSCRAMMs, which are important for adherence to the extracellular matrices of mammalian cells. Finally, the activation of lipase (Sal-1 and Sal-2) precursors by proteases secreted by *S. aureus* and the proteolytic processing of staphylococcal nucleases (Gotz et al. 1998; Suciu and Inouye 1996; Davis et al. 1977) might contribute to *S. aureus* dispersal and colonisation of new sites.

*S. epidermidis*, a common bacterial coloniser of mammalian skin, also produces exoproteases, namely cysteine (Ecp) and serine (Esp) proteases with relatively low substrate specificity (Dubin et al. 2001). The cysteine protease from *S. epidermidis* possesses the ability to cleave fibrinogen and fibronectin (Oleksy et al. 2004). Although Ecp and/or Esp have not been reported to contribute to *S. epidermidis* detachment from biofilm, these findings collectively indicate a significant contribution of proteases to bacterial dissemination via proteolytic inactivation of adhesive molecules.

The commensal lifestyle of *S. epidermidis* is partly achieved by the expression of the extracellular neutral metalloprotease SepA, which efficiently inactivates anionic AMP dermcidin (Lai et al. 2007). As part of the human skin microflora, *S. epidermidis* plays a probiotic function by preventing colonisation of more pathogenic bacteria such as *S. aureus*. One can argue that the SepA protease-mediated proteolytic degradation of AMP, a major determinant of innate host defence, creates a safe niche for dermicidin susceptible pathogens. Nevertheless, no evidence exists showing that *S. epidermidis* proteases facilitate the colonisation of the skin by other microorganisms in vivo.

*S. epidermidis* is a good example of the dependency of successful bacterial colonisation of the skin on the deterrence of AMP bactericidal activity. In the skin, AMPs are produced mainly by keratinocytes, neutrophils, sebocytes or sweat glands and are either expressed constitutively or after an inflammatory stimulus (Gallo and Nakatsuji 2011). In addition to exerting strong bactericidal potential by disrupting bacterial cell membranes, they can also act as potent immunomodulators. The function of defensins and cathelicidins, the best-characterised AMPs, is potentiated by AMPs derived from complement (Frick et al. 2006; Nordahl et al. 2004), haemoglobin (Mak 2008), serine proteases (Kasetty et al. 2011) or kininogen (Nordahl et al. 2005). Throughout evolution, microorganisms have developed many strategies to disable AMPs, one of them being proteolytic degradation. Despite the finding that AMPs are relatively resistant to proteolytic degradation, many skin pathogens produce proteases that can degrade human cathelicidins. Aureolysin, a metalloprotease of *S. aureus*, cleaves and inactivates LL-37, which might be the reason that highly proteolytic *S. aureus* strains are resistant to cathelicidin (Sieprawska-Lupa et al. 2004). *S. aureus* is not the sole pathogen that has evolved such a strategy. *S. pyogenes* is also well protected against LL-37 through an intricate mechanism involving streptopain (SpeB) expression. SpeB, an extracellular enzyme, is concentrated in the proximity of the bacterial cell membrane in a complex with an α-2-macroglobulin (α_2_M) immobilised on the streptococcal cell surface by interacting with the GRAB protein. Despite being entrapped by α_2_M, the enzyme is still proteolytically active against peptides, including LL-37. To reach the cell membrane, LL-37 must penetrate a layer of the α_2_M-streptopain complex coating the bacterium where it is proteolytically degraded, allowing *S. pyogenes* to resist AMPs. Indeed, SpeB has unambiguously been demonstrated to contribute to streptococcal resistance against LL-37 in vivo in patients with severe *S. pyogenes* infections of soft tissue (Johansson et al. 2008). Similarly, the colonisation of chronic ulcers by *P. aeruginosa*, *E. faecalis* and *P. mirabilis* might be related to the proteolytic inactivation of LL-37 by elastase, gelatinase and metalloprotease (ZapA), respectively (Schmidtchen et al. 2002). The degradation of LL-37 by a subtilisin-like serine protease of *Finegoldia magna* (formerly *Peptostreptococcus magnus*), a commensal bacterium colonising human skin and mucous membranes, has also been documented (Karlsson et al. 2007).

The evolutionary adaptation of *S. aureus* and *S. epidermidis*, inhabitants of healthy human skin, to this organ chemical defence is manifested by sensing dermicidin. In response to dermicidin, the extracellular proteolytic activity of staphylococci is enhanced, resulting in proteolytic degradation of dermicidin and bacterial resistance to this antibacterial peptide (Lai et al. 2007). Since bacterial proteases can inactivate the most important chemical antimicrobial barrier in skin, namely AMPs, further invasion and penetration into the deeper skin layers is highly likely. Nevertheless, it must be stressed that none of the bacteria can penetrate intact skin and cause infection unaided. However, once the integrity of the epidermis is compromised by cuts, wounds, burns, abrasions or bites, bacterial proteases released from colonising pathogens can significantly deter the immune system and lead to further tissue damage and to bacterial dissemination and infection.

### Tissue damage/injury

Bacterial proteases possess a propensity to destroy host tissue by two distinct mechanisms: direct breakage of the skin barrier, which occurs by cleavage of structural proteins in the skin, or indirect damage, which occurs following an excessive induction of the inflammatory response.

#### Direct mechanism of proteases action

*S. aureus* secretes exfoliative toxins, known as epidermolytic toxins (ETs), which cause blisters in bullous impetigo and staphylococcal scaled-skin syndrome (SSSS). Staphylococcal scalded skin syndrome is a disease that predominantly affects infants and is characterised by the loss of superficial skin layers, dehydration and secondary infections. The toxin is a serine protease of ~30 kDa characterised by a narrow substrate specificity. ET recognises and cleaves only desmoglein 1, a desmosomal cadherin that mediates cell–cell adhesion (Amagai et al. 2002; Hanakawa et al. 2004). The target protein, desmoglein 1, is recognised both through an interaction at the classical P1 site and via additional features in the tertiary structure, located away from the hydrolysed peptide bond. Disruption of desmoglein 1 by ETs in the deep layers of skin can be compensated by another desmoglein, desmoglein 3. Therefore, exfoliation only occurs in the SG, in which desmoglein 3 is not present. Accordingly, the hydrolysis of desmoglein 1 (but not that of other desmogleins) by ETs has been demonstrated experimentally both in vitro and in vivo, thus elucidating the mechanism of ET-induced epidermolysis (Amagai et al. 2000; Hanakawa et al. 2002). The toxin can spread through the bloodstream and therefore not all lesions are infected. Overall, the destruction of the epidermal barrier facilitates efficient progression of the infection. Three exfoliative toxins (ET), namely A, B and D, encoded by *eta*, *etb* and *etd*, respectively, have been identified (Lee et al. 1987). ETA-producing strains dominate Europe, USA and Africa (de Azavedo and Arbuthnott 1981). Expression of both *eta* and *etb* is regulated by *agr* (Sheehan et al. 1992).

The *S. aureus* extracellular serine protease, glutamylendopeptidase SspA, commonly referred to as V8 protease, is also regulated by the *agr* system. SspA preferentially cleaves peptide bonds with glutamate (and aspartate, to a lesser extent) at the carboxy-terminal side (Ono et al. 2010). Interestingly, the SspA protease shows sequence similarity to exfoliative toxins and shares a similar specificity of glutamate-specific cleavage (Dubin 2002). Recently, SspA has been reported to impair the epidermal permeability barrier in nude mice by disturbing the structure of the SC but does not cause epidermal hyperproliferation and inflammatory infiltration. Evaluation of SspA-induced injury in hairless mice with normal immune systems has confirmed results obtained when nude mice are used as a model of infection (Hirasawa et al. 2010). Furthermore, analysis by scanning electron microscopy has shown a reduced abundance of adhesive corneocytes on the skin of mice on which protease is applied. Based on the high degree of similarity between the primary and tertiary SspA structures and the preference of exfoliative toxins for Glu-Xaa peptide bonds, one can speculate that the epidermal permeability is the consequence of desmoglein 1 cleavage in corneodesmosomes. Disruption of the epithelial barrier by *S. aureus* extracellular protease compromises the protective functions of the skin by allowing the entry of allergens and microorganisms. A similar effect has been reported for a cysteine protease of house-dust mites, which impairs the epidermal permeability barrier, thus enhancing IgE and IgG responses to penetrating allergens (Kato et al. 2005; Kikuchi et al. 2006; Nakamura et al. 2006). This can additionally aggravate allergic reactions attributable to mast cell activation by infection (McAlpine et al. 2011). Notably, in this context, *S. aureus* can subvert the extracellular antimicrobial activity of mast cells by promoting its own internalisation by these cells (Abel et al. 2011).

Once proteolytic enzymes produced by skin pathogens disrupt the keratynocyte barrier, the underlying tissue layers can be penetrated by microorganisms and their products. Recently, the translocation of *S. pyogenes* (Group A streptococcus, GAS) through damaged skin has been documented as being facilitated by streptolysin S. This process is accompanied by the cleavage of transmembrane junctional proteins, including E-cadherin. Interestingly, streptolysin acts in concert with calpain, the host cysteine protease (Sumitomo et al. 2011).

Elastin is one of the connective tissue components of the dermis layer. *S. aureus* secretes two papain-like cysteine proteases, namely staphopain A (ScpA) and staphopain B (SspB). ScpA exerts elastinolytic activity comparable with neutrophil elastase, which might explain the destruction of connective tissue during staphylococcal infection (Potempa et al. 1988). Furthermore, recent studies indicate that the presence of staphopains at concentrations as low as 10 nM can also degrade collagen (Ohbayashi et al. 2010). *S. epidermidis*, a predominant inhabitant of human skin, express a cell-wall-associated cysteine protease Ecp. A homology search by using the N-terminal sequence has revealed sequence similarity to *S. aureus* ScpA and SspB. Consistent with this, the Ecp protease also possesses elastinolytic activity, which might contribute to the invasiveness and pathogenic potential of *S. epidermidis* (Oleksy et al. 2004).

Elastase, the major metalloproteinase expressed by *P. aeruginosa*, degrades proteins on the surface of skin-derived fibroblasts. Moreover, this enzyme can inhibit fibroblast cell growth. These effects, in conjunction with *ex vivo* data showing that elastase is present in the fluids of wounds infected with *P. aeuginosa*, suggest that bacterial proteinases play a pathogenic role in chronic ulcers (Schmidtchen et al. 2003).

Bacterial enzymes also contribute indirectly to tissue injury by intercepting the functions of tightly regulated host enzymes. Alternatively, they can release the activity of endogenous proteases from the control exerted by proteinase inhibitors, including cystatins and elafin (Vincents et al. 2007; Guyot et al. 2010). Cystatins regulate skin barrier formation by inhibiting the activity of cathepsin V, cathepsin L and legumain, thereby controlling transglutaminase 3 processing. Infringement of this pathway leads to abnormal SC and hair follicle formation and a severe malfunction of the skin barrier probably contributes to the dysregulation observed in inflammatory dermatoses (Cheng et al. 2009). *S. aureus* cysteine proteases ScpA and SspB are not inhibited by human cystatins; instead, extracellular cystatins C, D and E/M are hydrolysed by both staphopains (Vincents et al. 2007). Thus, the normal activity of the cystatins is down-regulated, indicating that bacterial enzymes can alter the host protease-inhibitor balance. Moreover, the inactivation of cystatins can lead to the enhancement of cathepsin activity, which in turn can inactivate AMPs. Despite a broad range of in vitro and *ex vivo* results demonstrating the pathogenic potential of staphopains, their role as important staphylococcal virulence factors in vivo still needs to be demonstrated by using appropriate animal models of *S. aureus* infection.

#### Indirect mechanism of proteases action

The host defence system has to cope with various microorganisms to obtain equilibrium, otherwise an exacerbated inflammatory response can lead to tissue injury. Penetration of bacteria through the skin barrier mobilises a broad range of host defence mechanisms, such as professional phagocytes, the complement system and cytokines. Since bacteria proteases possess the ability to inactivate, derail or interfere with these defence mechanisms, they are considered to be potential virulence factors.

##### Professional phagocytes

Cellular defence involves neutrophils and macrophages that infiltrate subepithelial connective tissues. The critical step that precedes bacterial engulfment is the recognition of pathogens. The efficiency of this step is enhanced by bacteria opsonisation with specific antibodies and/or complements. IdeS is a cysteine protease of *S. pyogenes* that allows GAS to evade antibody-mediated phagocytosis by cleaving IgG at the lower hinge region. Simultanously, IgGs captured via their Fc region by immunoglobulin-binding proteins, streptococcal M or M-like proteins on bacterial surfaces are protected from proteolysis; this allows the formation of a host-like coat by IgG molecules (Su et al. 2011). Interestingly, cystatin C, a cysteine protease inhibitor, acts as a cofactor that accelerates IgG cleavage by IdeS (Vincents et al. 2008). Another strategy aimed at professional phagocytes involves the use of streptopain (streptococcal pyrogenic exotoxin B, SpeB) by *S. pyogenes* (Nelson et al. 2011). SpeB internalised by neutrophils can cause mitochondrial damage manifested by a decrease in dehydrogenase activity and a loss of membrane potential after r-SpeB treatment. Although the incubation of neutrophils with the wild-type strain, the *speB* mutant, or the r-SpeB protein does not affect the total number of viable cells, one can argue that professional phagocyte functions are disabled (Chiang-Ni et al. 2006).

A significant reduction in the number of functional phagocytes at infection sites can be potentially induced by SspB, the cysteine protease of *S. aureus*. This effect is exerted by selective cleavage of CD11b on phagocytes, which rapidly acquire the features of a dead cell (Smagur et al. 2009a). Furthermore, exposure of phagocytes to SspB blocks the phagocytosis of *S. aureus* by neutrophils, represses their chemotactic activity and induces extensive non-phlogistic clearance of SspB-treated cells by macrophages. The latter effect occurs by the proteolytic degradation of CD31, which constitutes a repulsive “do not-eat-me” signal on the surface of leucocytes (Smagur et al. 2009b). Collectively, this may lead to the depletion of functional neutrophils at infection sites, thus facilitating staphylococcal colonisation and spreading in subepithelial tissues.

The intracellular persistence of pathogens in both professional phagocytes and non-phagocyting cells is supported by the expression of bacterial proteases. An *S. aureus* metalloprotease, aureolysin, contributes to survival within macrophages (Kubica et al. 2008). Following phagocytosis by human neutrophils, aureolysin is strongly expressed by engulfed *S. aureus*, thereby confirming enzyme production during host-pathogen interaction (Burlak et al. 2007). Additionally, in vivo, SpeB appears to contribute to the intracellular survival of *S. pyogenes* in macrophages during acute invasive infections (Thulin et al. 2006). Together, the data make it clear that bacterial proteases can play a role in protecting bacteria from phagocytes in skin layers infiltrated by these immune cells.

##### Complement system

The complement system participates in the immune recognition of foreign antigens, many of which might penetrate the skin by physical injury or transcutaneous adsorption. Pathogens target and subvert the complement system by degrading or binding complement components to prevent the activation of the pathway. Since the C3 and C5 convertase complexes play a pivotal role in activating the complement system, they are the major targets of many bacterial proteases (Potempa and Pike 2009).

Recent data indicate that *S. aureus* aureolysin disables complement activation by inhibiting the deposition of C3b on the bacterial surface and the release of the chemoattractant C5a, practically paralysing all complement-dependent antibacterial functions. This occurs because aureolysin cleaves C3, the central complement protein, generating C3b, which is further degraded by host factors. Thus, aureolysin acts in synergy with host regulators to inactivate C3, effectively dampening the host immune response (Laarman et al. 2011). Another skin pathogen, *S. pyogenes*, prevents the formation of the C5 convertase complex, since SpeB efficiently degrades C3 (Kuo et al. 2008; Terao et al. 2008). SpeB protease also cleaves properdin, which stabilises the formation of the C5 convertase complex through an alternative complement activation pathway (Tsao et al. 2006). Finally, a specific *S. pyogenes* enzyme, C5a peptidase (Wexler et al. 1985), disables the complement-dependent influx of neutrophils to sites infected with *S. pyogenes*. C5 peptidase strongly contributes to *S. pyogenes* pathogenicity (Ji et al. 1996, 1997; Cleary et al. 2004) and might be essential for protecting streptococci during skin infections. Interestingly, C5a peptidase expression is upregulated in bacteria by human serum (Gleich-Theurer et al. 2009).

##### Cytokine and chemokine network

Chemokines are a large superfamily of cytokines that provide chemotactic activity for immune cells. Modulation of their activity can occur upon proteolytic processing at both N- and C-termini. Some bacterial species, such as *S. pyogenes*, use proteases to exploit this mechanism. The *S. pyogenes* protease ScpC degrades interleukin-8 (IL-8), a chemokine that mediates neutrophil transmigration and activation. Bacteria expressing ScpC overcome immune clearance by preventing the recruitment of neutrophils to soft tissue infection in mice (Sjölinder et al. 2008). *Finegoldia magna*, a commensal that is also associated with skin and soft tissue infections, express subtilisin-like extracellular serine protease (SufA). SufA is associated with the bacterial cell surface but is also released in substantial amounts during bacterial growth. SufA has been found to cleave and inactivate the CXC chemokine MIG/CXCL9 efficiently, indicating that the enzyme promotes *F. magna* survival and colonisation (Karlsson et al. 2007). Recently, serine proteases derived from *S. aureus* have been found to initiate and maintain the inflammatory response by modulating IL-8 synthesis and nuclear factor kappa B (NFκB) activation (Rudack et al. 2009). A similar effect, i.e. induction of IL-8 secretion, has also been documented for *P. aeruginosa* exoprotease LepA (Kida et al. 2008).

Chemerin is a ligand for CMKLR1, a seven transmembrane G-protein-coupled receptor. Chemerin circulates as an inactive precursor (prochemerin) in blood but its cytokine expression (specific mRNA) has also been detected in skin (Wittamer et al. 2003). Maturation of chemerin into an active cytokine requires proteolytic processing. Recently, active chemerin has been demonstrated to be generated by the secreted cysteine protease SspB of *S. aureus* (Kulig et al. 2007). In skin infected with *S. aureus*, proteolytically processed chemerin might therefore contribute to the pathological inflammatory response by attracting selected immunoregulatory antigen-presenting cells, such as immature plasmacytoid dendritic cells (Skrzeczyńska-Moncznik et al. 2009) and macrophages. The potential in vivo role of staphopain in chemerin activation correlates with the finding that *S. aureus* has been implicated as an aggravating factor in psoriasis (Kulig et al. 2007). Consistent with this, high levels of active chemerin have been found in the skin of psoriatic patients (Skrzeczyńska-Moncznik et al. 2009).

##### Cell signalling

The protease-activated receptors (PARs) are a family of four G-protein-coupled receptors that are activated upon enzymatic cleavage at their N-termini by specific serine proteases. The exposed tethered ligand domains bind to and activate the cleaved receptors. Four human members of the family are known: PAR1–4. PAR-1, PAR-3 and PAR-4 are activated physiologically by thrombin, whereas PAR-2 activation is associated with a proinflammatory response. PAR-2 is highly expressed in keratinocytes and might play an important role in cutaneous inflammation. PARs are targeted by many bacteria-derived proteases (Imamura and Potempa 2011), including enzymes produced by skin pathogens. LepA, a secreted protease from *P. aeruginosa*, signals through PAR-1-, PAR-2- and PAR-4-specific proteolysis and activates the NFκB pathway (Kida et al. 2008). Simultaneously, LasB, an elastynolytic metalloprotease from the same bacterium, antagonises the effect of LepA by degrading and inactivating the receptor (Dulon et al. 2005). *Serratia marcescens*, a rare cause of skin infections, expresses serralysin, which cleaves PAR-2 on HeLa cells. This leads to the activation of critical transcription factors AP-1, C/EBPβ and NFκB (Kida et al. 2007). Recently, exogenous proteases from *Propionibacterium acnes* have been demonstrated to elicit cellular responses via PAR-2. Activation of PAR-2 on keratinocytes has been shown to lead to the induction of proinflammatory cytokines, AMPs and matrix metalloproteinases (Lee et al. 2010).

##### Kallikrein/kinin system

The contact system is an integral part of skin innate immunity, which, once activated, delivers bactericidal peptides derived from kininogens, entraps bacteria and stimulates professional phagocytes. Furthermore, kinins, such as bradykinin (BK) and its metabolite, desArg9BK, can act as co-mitogens in cellular proliferation and as proinflammatory factors, which are important for vascular permeabilisation and pain propagation (Schremmer-Danninger et al. 1999).

Initially, vascular leakage (VL) supplies antimicrobial factors (e.g. antibodies, complement factors) and causes blood leucocyte infiltration, contributing to pathogen elimination. An excessive inflammatory reaction, a consequence of serious tissue damage, can be induced by bacterial proteases, which play a crucial role in the uncontrolled generation of proinflammatory mediators. Activation of the kallikrein-kinin system is initiated by Hageman factor (HF) activation, followed by the production of kallikrein from plasma prekallikrein (PK). High molecular weight kininogen is the substrate for plasma kallikrein, which releases BK. On the other hand, kallidin is generated from low-molecular weight kininogen by tissue kallikrein (Chao et al. 2010). Kallidinis are ultimately converted to BK (Cochrane and Griffin 1982; Bhoola et al. 1992).

Bacterial proteases are potent activators of contact system zymogens (Herwald and Potempa 2011). Alkaline proteinase and elastase from *P. aeruginosa* and metalloproteases from *V. vulnificus* activate HF and PK (Molla et al. 1989); however, only the latter has been described in the human (Miyoshi and Shinoda 1992). *Aeromonas sobria*, a facultative anaerobe that can cause skin infections, expresses serine proteases (ASP) that induce vascular leakage activity by specifically activating human PK (Jones and Wilcox 1995; Imamura et al. 2006).

The release of kinins requires the cleavage of kininogen at two peptide bonds; this can be achieved directly by bacterial proteases without PK or HF activation. Bacterial proteases, such as staphopains from *S. aureus* or streptopain from *S. pyogenes*, are important virulence factors, since they can potently release kinins from kininogens (Imamura et al. 2005; Herwald et al. 1996). Proteolytically active ScpA has been found to induce VL in a BK B(2)-receptor-dependent manner in guinea pig skin. This effect is augmented by SspB, which, by itself, has no VL activity (Imamura et al. 2005). The enhancing effect of SspB is attributable to the release of a new kinin that contains an amino terminus that is extended by three amino acid residues. Furthermore, plasma leakage spreads in subcutaneous tissue, since ScpB degrades elastin and other extracellular matrix proteins (Potempa et al. 1988). A similar mode of action has been reported for serine protease ASP secreted by *A. sobria*, which induces VL activity mainly in a BK B(2) receptor-dependent manner in guinea pig skin. ASP produces more VL activity directly from human low-molecular-weight kininogen than from high-molecular-weight kininogen (Imamura et al. 2006). Furthermore, ASP produces far more desArg-BK than BK from kininogens. DesArg-BK binds to the B1 receptor and responses mediated by this receptor are upregulated by lipopolysaccharide or inflammatory cytokines in animal and human tissues (Marceau et al. 1998). Collectively, the data clearly show that bacterial proteases play an important role in the amplification of kinin generation, which can be associated with the pathophysiology of infectious diseases. Such tactics can facilitate systemic dissemination of a pathogen from the initial site of colonisation.

## Concluding remarks

Taken together, the above data clearly reveal that proteases secreted by pathogenic bacteria that colonise damaged skin play a role in the invasion of deeper skin layers and contribute to the development of a local inflammatory reaction. This function is in stark contrast to proteases of skin commensal microorganisms, which use their proteases to maintain an equilibrium with host defences and homeostasis of the colonised skin.
